# Protective effect of 14-3-3 antibodies on stressed neuroretinal cells via the mitochondrial apoptosis pathway

**DOI:** 10.1186/s12886-015-0044-9

**Published:** 2015-06-27

**Authors:** Katharina Bell, Corina Wilding, Sebastian Funke, Norbert Pfeiffer, Franz H. Grus

**Affiliations:** Experimental Ophthalmology, Department of Ophthalmology, University Medical center of the Johannes Gutenberg University, Langenbeckstraße 1, 55131 Mainz, Germany

**Keywords:** Autoantibodies, Glaucoma, Neurodegeneration, Natural autoimmunity, Neuroprotection

## Abstract

**Background:**

Previous studies demonstrate changes of autoantibody concentrations against retinal and optic nerve head antigens in the serum of glaucoma patients in comparison to healthy persons. These antibodies belong to the natural autoimmunity. Previous studies showed up regulated, but also significantly down-regulated autoantibody levels. These antibodies have the ability to influence protein profiles of neuroretinal cells and possibly hold neuroprotective potential, as we have been able to demonstrate before. Aim of this study was to analyse the serum and antibody effect of glaucoma patients on neuroretinal cells in more detail and also determine the impact of antibodies found down-regulated in glaucoma patients on the pathogenesis of the neurodegenerative disease glaucoma.

**Methods:**

Neuroretinal cells (RGC-5) were incubated with serum either from glaucoma patients or healthy controls for 24 h. Mass spectrometric analysis was performed after cell lysis. Furthermore the neuroretinal cells were preincubated with different and concentrations of 14-3-3 antibodies (0.005, 0.1, 0.5, 1, 5 and 10 μg/ml) and then stressed with H2O2, staurosporine or glutamate. Viability tests were performed with crystal violet and ROS tests with DCFH-DA. Antibody location in the cell after antibody incubation was performed with immunoccytochemical methods. Additionally mass spectrometric analysis was performed with the cells after antibody incubation.

**Results:**

Protein expression analysis with Maldi-Orbitrap MS showed changes in the expression level of regulatory proteins in cells incubated with glaucoma serum, e.g. an up-regulation of 14-3-3 and a down-regulation of Calmodulin. After preincubation of the cells with anti-14-3-3 antibody and stressing the cells, we detected an increase in viability of up to 22 % and a decrease in reactive oxygen species (ROS) of up to 31 %. Proteomic 1 analysis involvement of the mitochondrial apoptosis pathway in this protective effect and immunohistochemical analysis showed an antibody uptake in the cells.

**Conclusion:**

We found significant effects of serum antibodies on proteins of neuroretinal cells especially of the mitochondrial apoptosis pathway. Furthermore we detected a protective potential of antibodies down-regulated in glaucoma patients. The changed autoantibodies belong to the natural autoimmunity. We conclude that changes in the natural autoimmunity of patients with glaucoma can negatively impact regulatory functions.

**Electronic supplementary material:**

The online version of this article (doi:10.1186/s12886-015-0044-9) contains supplementary material, which is available to authorized users.

## Background

The pathogenesis of neurodegenerative diseases is often poorly understood. Neurodegenerative diseases are characterised by progressive nervous system dysfunction and an accompanying atrophy of the affected central or peripheral nervous system [1]. As in other neurodegenerative diseases, such as amyotrophic lateral sclerosis, Alzheimer’s or Parkinson disease, glaucoma leads to the apoptotic loss of one specific neuron population, the retinal ganglion cells (rgc) [2]. An atrophy of central structures such as the lateral geniculate nucleus [3] can also be found. With an estimated prevalence of at least 60 million cases worldwide [4], glaucoma can be counted to the list of the most common neurodegenerative diseases [5].

This heterogeneous group of eye diseases, with a still unknown pathogenesis, demonstrates with a progressive loss of retinal ganglion cells (rgc), optic nerve degeneration and visual fields loss, finally leading to blindness [6]. 2.65 % of the world’s population above the age of 40 suffers from glaucoma [7]. The major risk factor for developing glaucoma found in approximately 70 % of the patients is an increased intraocular pressure (IOP) [8, 9].

Other pathogenesis factors leading to apoptosis of rgc [10, 11] such as elevated levels of reactive oxygen species (ROS) [12, 13] or elevated glutamate levels are discussed [14, 15]. Furthermore, there is strong evidence that an immunologic component is involved in glaucoma pathogenesis. Altered autoantibody levels in the serum of glaucoma patients e.g. against heat shock protein (hsp) 60 [16], alpha crystallin and hsp27, gamma enolase [17] and glycosaminoglycans as well as against human retinal antigens, such as against cellular retinaldehyde-binding protein and retinal-S-antigen [18, 19] have been demonstrated. Interestingly, the studies were not only able to detect higher concentrations of different autoantibodies in glaucoma patients, but also lower concentrations of many autoantibodies in comparison to healthy people [20]. Many of the serum immunoglobulins in healthy people belong to the so called natural autoimmunity [21, 22]. These autoantibodies do not cause diseases and in contrast are considered as regulatory factors [23]. In general it is known that up-regulated autoantibodies can be auto-aggressive and lead to pathogenic conditions, such as the antibody against postsynaptic nicotinic acetylcholine receptor in patients suffering from myasthenia gravis [24]. The role of the down-regulated autoantibodies found e.g. in glaucoma patients, but also in patients suffering from other neurodegenerative diseases, such as Alzheimer’s disease [25], so far is not known. We assume that the down-regulation of some of the antibodies can lead to changes in the regulatory function of these antibodies and therefore could be involved in the pathogenesis of the neurodegenerative disease glaucoma.

The aim of this study was to investigate the induced effect of glaucomatous serum and an antibody found down-regulated in glaucoma patients on viability, reactive oxygen levels (ROS) as well as the proteomics of neuroretinal cells. In previous studies we were able to demonstrate that the antibodies of glaucoma patients in general have a large influence (59 %) on the protein profiles of neuroretinal cells [26]. Therefore we analysed the changes of proteins and their pathways in more detail. Additionally we enlighten whether down-regulated antibodies could have an impact on the disease glaucoma.

In summary we demonstrate that serum of glaucoma patients can change intracellular concentrations of important regulatory proteins such as the protein kinase inhibitor 14-3-3, which has a major regulatory function on the MAPK/ERK pathway, or Calmodulin. Furthermore we were able to show that the antibody against 14-3-3, which is down-regulated in glaucoma patients (p < 0.01) (see Additional file 1: Figure S1), has protective effects on stressed neuroretinal cells, documented by an increased viability and reduced ROS-level of the cells.

## Methods

### Reagents

The used reagents were purchased from Sigma Aldrich, St. Louis, MO, US if not stated otherwise. L-alanyl-L-glutamine was purchased from Biochrom AG (Berlin, Germany). The rabbit anti-14-3-3 abs for cell incubation and the secondary antibody goat-anti-rabbit IgG-H&L (FITC) were purchased from Abcam (Cambridge, UK), H_2_O_2_ and paraformaldehyde from Carl Roth GmbH (Karlsruhe, Germany), Staurosporine from Calbiochem (San Diego, CA), 70 % ethanol, formic acid, trifluoroacetic acid (TFA) and acetonitrile (ACN) from Merck (Darmstadt, Germany), 14-3-3 antibodys for immunohistochemical staining from Lifespan (Seattle, WA), the BCA Pierce Protein Assay kit from Fisher scientific (Waltham, MA), Trypsin from Promega (Mannheim, Germany), wheat germ agglutinin conjugate with tetramethylrhodamin (WGA) from Invitrogen (Karlsruhe, Germany), vectashield mounting medium with 4’,6-diamidino-2-phenylindole (DAPI) from Vector Laboratories (California, USA) and HPLC H_2_O from Applichem (Darmstadt, Germany).

### Serum samples

The serum was collected from patients suffering from POAG according to the classification of the guidelines of the European glaucoma society as well as from healthy volunteers after given written informed consent. Furthermore, patients suffering from autoimmune diseases or other neurodegenerative diseases were excluded from the study. The studies were performed in accordance with the Declaration of Helsinki on medical research involving human subjects. The samples were age matched. Ethics approval: No: 837.219.07 (5754); Ethics committee of the Landesärztekammer Rhineland-Palatinate.

### Cell culture

RGC-5 cells, a neuroretinal cell line of mouse origin, provided by Dr. Neeraj Agarwal, were used as model for retinal cells of neuronal origin [27]. They were grown in 75 culture flasks in Dulbecco’s modified eagle medium supplemented with 10 % fetal calf serum (FCS), 100 U/ml penicillin, 100 U/ml streptomycin and 4 % L-alanyl-L-glutamine. The cells were cultivated in a humidified incubator at 37 °C with 95 % air and 5 % CO_2_ and were passaged when they reached a confluence of 80 %.

### Cell treatment with different serum types

250 000 RGC-5 cells were transferred to culture dishes with an 82.7 mm inner diameter. After 24 h the cells were incubated with medium containing 5 % FCS and either 5 % serum from healthy people or 5 % serum from POAG patients (n in each experimental group: 8). Each individual serum was used for an individual run. The cells were incubated with the different serum types for 24 h. After 24 h the medium was discarded and the cells were washed twice with phosphate buffered saline (PBS). Cell lysis was performed and the detached cells were transferred to an Eppendorf tube with lysis buffer [Urea 9.5 M, Chaps 2 %, DTT 1 % + proteinase inhibitor mix (P 1860)]. Furthermore an ultrasonic pulse echo instrument [Labsonic®M (Sartorius)] was used to perform additional cell lysis. Protein concentration of the samples was determined with the method of Lowry [28]. After the protein concentration was determined, a sample-pool for each experimental group was created with a total amount of 80 μg protein. The pooled samples were separated with a 1D SDS gel. Each lane was divided into 15 pieces and digested with trypsin in order to measure the peptide profile with Maldi- Orbitrap MSMS.

### Protein profiling with Maldi- Orbitrap MSMS

The protein profiles were analysed with Maldi- LTQ Orbitrap XL using Maldi-steel targets. The samples were dried in a concentrator and acidified with 0.1 % TFA. C-18 ZipTips (Millipore, Billerica, MA) were used to purify the samples and the peptides were eluted directly onto a Maldi Target with 40 % ACN and 60 % ACN. Measurements were performed according to the manufacturers’ protocol.

### Protein profiling with capillary LC-ESI-MSMS

The protein profiles were analysed with capillary LC-ESI-MSMS using a C-18 pre-column (30 mm x 0.5 mm) and a C18 analytical column (150 mm x 0.5 mm, both Thermo Scientific,). A Rheos Allegro HPLC Pump (Thermo Scientific) was the solvent delivery system. The pump flow rate was 200 μl/min, and reduced to a column flow of 10 μl/min (M-472 graduated microsplit valve (Upchurch, Scientific, USA). With two running buffers (A (98 % H_2_O, 1.94 % ACN, 0.06 % methanol, 0.05 % TFA) + B (95 % ACN, 3 % methanol, 2 % H_2_O, 0.05 % TFA) a linear gradient of 80 min was performed (0–47 min: 0–100 % B, 47–49 min: 100 % B, 49–58: 100 %–0 % B, 58–80 min: 0 % B). Mass spectra were obtained using an LTQ OrbitrapXL.

### Cell treatment with 14-3-3 antibodies and different stress factors

**Fig. 1:**
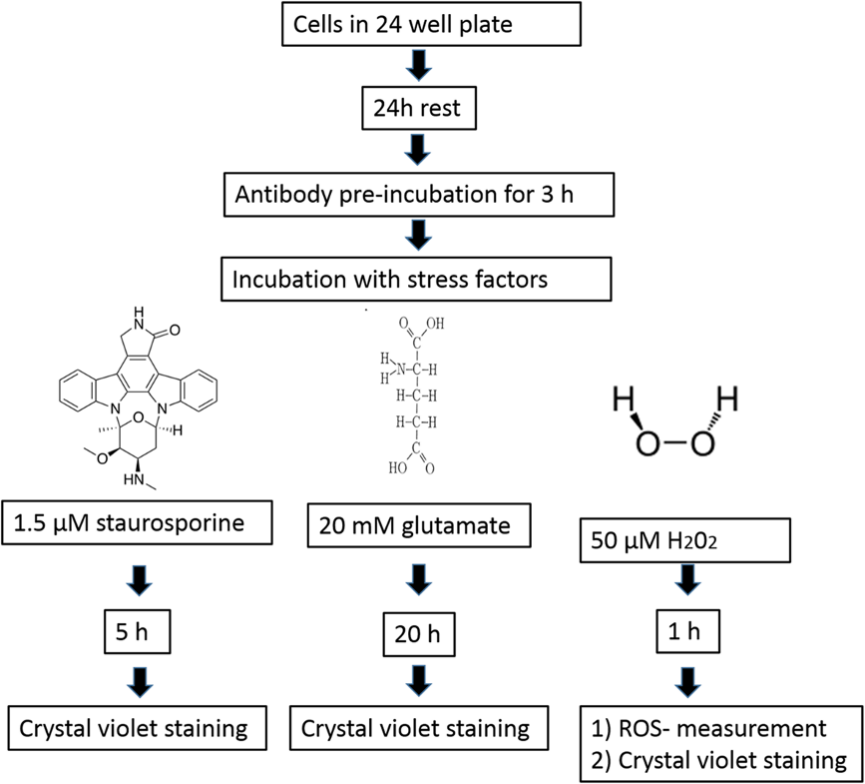
Experimental setup for RGC-5 cells incubated with 14-3-3 ab. The cells were seeded in 24 well plates and were let to rest for 24 h. Then antibody preincubation of the cells with different antibody concentrations was performed for 3 h. The control cells not incubated with the antibody were incubated with normal medium for the same amount of time. After 3 h the medium containing the antibodies or the control medium was replaced with medium containing one of the stress factors (glutamate, staurosporine or H_2_O_2_). Depending on the stress factor, the incubation time varied. After stressing the cells, viability tests with crystal violet staining was performed with all cells, and ROS level measurements were performed in the cells stressed with H_2_O_2_

RGC-5 cells were seeded in 24 well plates. Depending on the stress factor and therefore the overall incubation time, the cells were either seeded with 45000 cells per well (for the experiments with H_2_O_2_ and staurosporine) or 40000 cells per well (experiments with the stress factor glutamate). The cells were preincubated with different concentrations of chicken polyclonal anti 14-3-3 sigma antibodies (0.005, 0.1, 0.5, 1, 5 and 10 μg/ml) for 3 h. As an additional control group the cells were incubated with a non-retina specific antibody against myoglobin. To induce apoptosis the cells were stressed with staurosporine (1.5 μM for 5 h) or glutamate (20 mM for 24 h). Furthermore oxidative stress was induced by incubating the cells with 50 μM H_2_O_2_ for 1 h (n in each experimental group = 4). We used this amount of H_2_O_2_ because test showed that we were able to detect a rise in ROS without a loss of viability of the cells using this concentration. Subsequently cell viability tests and ROS measurements were performed. Figure 1 shows an overview of the experimental setup.

### Cell viability test

Cell viability was assessed with crystal violet staining. The cells were fixed with 3 % paraformaldehyde (15 min) and rinsed with PBS. Subsequently the cells were stained with 0.1 % crystal violet solution for 20 min. Excess stain was removed by washing the plates with distilled water three times. The bound stain of the viable cells was resolved in 70 % ethanol for 3 h. The supernatants were read with the Multiscan ascent plate reader (Thermo scientific) at 570 nm. The absorption was expressed as a percentage of the control cells, which were only treated with the stress factor. An unpaired student *t*-test was used to compare the data obtained and was realized with Statistica (Statsoft, Tulsa, Oklahoma, USA). A p value < 0.05 was significant and a p value < 0.01 was declared highly significant.

### ROS-test

To quantify ROS we used 2’,7’-dichlorodihydrofluorescein-diacetate (DCFH-DA). Intracellular esterases’ and ROS convert the non-fluorescence stain 2’, 7’ dichlorodihydrofluorescein (DCFH) to the fluorescent stain dichlorofluorescein (DCF). Cells were loaded with 10 mM DCFH-DA in the incubation chamber for 15 min. Then the culture medium was replaced, to remove the unbound DCFH-DA. To generate ROS, 50 μM H_2_O_2_ was added. The fluorescence was measured by using the microplate reader fluoroscan ascent (Thermo scientific) with excitation/emission wavelengths of 485/538 nm. The absorption was expressed as a percentage of the control cells, which were only treated with 50 μM H_2_O_2_. The ROS- level was normalised by measuring the viability of the cells in the same well. An unpaired student *t*-test was used to compare the data obtained and was realized with Statistica.

### Immunocytochemical staining

RGC-5 cells were grown in μ-slide IV (Ibidi GmbH, Munich, Germany) and subsequently washed with PBS. Then the cells were fixed with 3 % paraformaldehyde for 15 min and incubated with 0.25 % Triton-X-100 for 12 min. After 3 wash steps with PBS, the cells were treated with 1 % bovine serum albumin for 20 min. Afterwards, the cells were incubated with 2 μg/ml rabbit polyclonal anti 14-3-3 sigma antibodies overnight, then gently washed 3 times with PBS and incubated with Goat polyclonal secondary antibody to rabbit IgG-H&L conjugated with FITC for 1.5 h. After 3 washing steps with PBS the cells were visualized with a fluorescence microscope (Leica Microsystem, Heidelberg, Germany). To investigate the antibody uptake in living cells, the cells were preincubated with 10 μg/ml rabbit polyclonal anti 14-3-3 sigma abs for 3 h and then washed with PBS to remove unbound antibodies. Controls were preincubated with medium not containing the polyclonal anti 14-3-3 sigma abs. The cells then were treated as described above and visualized with a Leica fluorescence microscope and using Lucia G/F software.

### Cell lysate preparation

For proteomic analysis the cells incubated with anti-14-3-3 antibody were grown in 60 x 15 mm cell culture dishes and incubated with 0.5 μg/ml chicken polyclonal anti 14-3-3 sigma antibodies. Cells were detached using cell dissociation solution (CDS) and lysed by freezing at −80 °C after adding 0.1 % Dodecyl-D-β- Maltosid with proteinase inhibitor. Additionally, the cells were treated with a sonication bath for 1 min. After centrifugation, the supernatant was used for determining the protein concentration by BCA Pierce Protein Assay kit.

### SDS PAGE separation and in-gel digestion

Protein separation was performed with a denaturing gel electrophoresis. Each lane was cut into 17 pieces, incubated with acetonitrile (ACN) and ammonium bicarbonate (AB) and dried in a concentrator. The pieces were digested with trypsin (0.7 μg Trypsin in 80 % HPLC H_2_O, 10 % ACN, 10 % AB) over night. The supernatant was collected and the remaining proteins were dissolved with an extraction buffer (38 % HPLC H_2_O, 2 % formic acid, 60 % ACN) for 30 min. The supernatants then were pooled, dried in a concentrator and acidified with 0.1 % trifluoroacetic acid. C-18 ZipTips were used to purify the samples. The samples were measured with capillary LC-ESI-MSMS as described above.

### Data processing

The obtained mass spectra measured with Maldi- Orbitrap MSMS were used for an identification and quantification of the proteins. Using Mascot search engine, the spectra were transferred to SwissProt database. The identification of the proteins was performed using Mus musculus as taxonomy and trypsin as digesting enzyme. This information is necessary for database to calculate the theoretical mass. Furthermore one missed cleavage was allowed. As MALDI was used, the charge state was set to 1+. The error window of the mass was set at 100 ppm and 0.8 Da. The normalisation and quantification of the peptides was performed with PSP (former P2M), our in-house proteomics pipeline software and transferred to Statistica software for quantification, as described before [29].

The obtained mass spectra measured with LC-ESI-MSMS were used for identification and quantification with Maxquant (Max Planck Institute of Biochemistry, Martinsried, Germany). The tolerance in mass precision for MS/MS was 20 ppm and 0.5 Da. The protein and peptide false discovery rate were set to 0.01 and the minimum peptide length was 6 amino acids. The evaluation was implemented with Ingenuity Pathway Analysis (IPA) Software. Only proteins with a 2-fold changed expression upon 14-3-3 sigma antibody treatment were included in the analysis. The statistical significance of each pathways was calculated by IPA using a Fisher Exact test *p* < 0.05.

## Results

### Changes in protein profiles of cells incubated with POAG serum

**Fig. 2:**
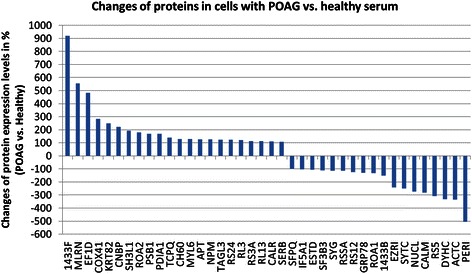
Changes of proteins in RGC-5 cells incubated with POAG serum versus healthy serum. Proteomic measurements of the pooled samples of the cells incubated with POAG serum or healthy serum were performed. Each pooled sample contained protein from each of the 8 samples of either the POAG or the healthy group. After identification of the proteins a quantification of the proteins of the cells incubated with POAG serum in comparison to healthy serum was performed. The graph shows the changes in %. The most up-regulated protein in the cells incubated with POAG serum was 14-3-3 eta. But also proteins such as Calmodulin (CALM) or zink-finger protein (CNBP) were significantly differently regulated. Proteins up- or down-regulated more than 2 fold (100 %) were considered to be statistically significant

We were able to detect complex protein profiles of RGC-5 cells incubated either with primary open angle glaucoma (POAG) or healthy serum. The measurements of the pooled samples (one of each group, consisting of a mixture of eight samples) with Maldi-Orbitrap MSMS showed 182 identified proteins of which 39 were significantly differently regulated (< −2 fold down-regulated or > 2 fold up-regulated) in cells incubated with POAG serum in comparison to healthy serum. Significant changes could be found throughout the cell, e.g. cytosolic proteins, as well as mitochondrial or nucleolus proteins. The most significantly up-regulated protein was identified as 14-3-3, a regulatory protein in eukaryotic cells. 14-3-3 protein was up-regulated 18 fold in cells incubated with POAG serum (Fig. 2, 14-3-3F_MOUSE). In comparison, Calmodulin (Fig. 2, CALM), a binding partner of 14-3-3 [30], was shown to be down-regulated nearly 6 fold (−5.6). Proteins, known from other glaucoma studies, such as zink-finger protein (CNPB), were detected to also be differently regulated in this study (Fig. 2).

### Protective effect of 14-3-3 antibodies

The effect of 14-3-3 antibodies (ab) on the cells was determined with crystal violet and DCFH-DA.

**Fig. 3:**
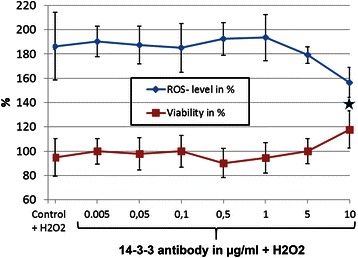
Protective effects of 14-3-3 antibody on RGC-5 stressed with H_2_O_2_. RGC-5 cells were preincubated with different concentration of 14-3-3 antibodies for 3 h and additionally stressed with 50 μM H_2_O_2_ for 1 h. This amount of H_2_O_2_ was used, as we did not want to produce a loss of viability through H_2_O_2_ our aim was to just increase ROS in the cells. The graph shows the ROS levels in % in the cells (blue line) as well as the viability (red line) of the cells after pre-incubation with different 14-3-3 antibody concentrations and stress with 50 μM H_2_O_2_ for 1 h. N in each experimental group is 4. Significantly decreased ROS levels (−31 %) were measured in the cells incubated with 10 μg/ml 14-3-3 antibodies. ROS-production was measured using DCFH-DA and expressed as percent of the control cells, which were only treated with H_2_O_2._ Significantly increased viability (+22 %) was measured for the cells incubated with 10 μg/ml 14-3-3 antibodies. Viability was measured using crystal violet and expressed as percent of the control cells additionally incubated with H_2_O_2_ (* = *p* < 0.05; **p < 0.01)

**Fig. 4:**
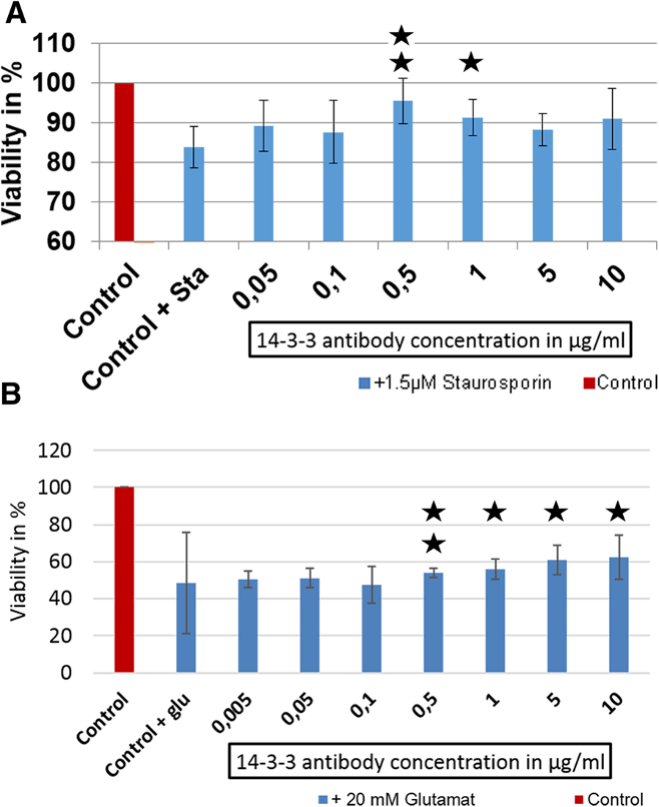
Viability of glutamate and staurosporine stressed RGC-5 upon 14-3-3 sigma antibody treatment. RGC-5 were preincubated with different 14-3-3 antibody concentrations and additionally stressed with 20 mM glutamate for 24 h, or 1.5 μM stauorsporine for 5 h. Cell viability was determined using crystal violet and expressed as percent of the control cells + the stress factor (glutamate or staurosporine) (* = *p* < 0.05; **p < 0.01). N in each experimental group is 4. **a**: Increased highly significant and significant cell viability of up to 7 % was demonstrated after the cells were preincubated with 0.5, 1 μg/ml 14-3-3 sigma antibodies and additionally stressed with staurosporine. **b**: Increased cell viability in a range of 0.05-5 μg/ml 14-3-3 antibodies were obtained after the cells were stressed with glutamate, whereby the result of 0.5 μg/ml is highly significant and of 1.5 μg/ml is significant. We were able to detect an increase of viability of up to 12 %

An increased cell viability of 22 % (*p* < 0.01) was measured after preincubation of the cells with 10 μg/ml 14-3-3 ab and additional stress with H_2_O_2_ (Fig. 3), as well as a significantly decreased ROS-production of 31 % (*p* < 0.01) (Fig. 3). We could indicate a significantly increased viability of 12 % (*p* < 0.01) when incubating the cells with 0.5 μg/ml 14-3-3 ab and of 7 % (*p* < 0.05) when incubating the cells with 1 μg/ml 14-3-3 ab and stressing with staurosporine (Fig. 4a).

The cells treated with glutamate showed a decreased viability of 51 %. We detected that cells incubated with 0.5, 1 and 5 μg/ml 14-3-3 antibodies showed an increased viability of up to 12 % (*p* < 0.01) in comparison to the control cells stressed with glutamate (Fig. 4b). In contrast, no positive or negative effect of the anti-myoglobin antibody, which served as a control, on the viability of the cells could be detected (see Additional file 2: Figure S2). Furthermore we could not detect any effect of the 14-3-3 antibody on non-stressed RGC-5 cells (see Additional file 3: Figure S3).

### Expression of 14-3-3 and 14-3-3 antibody binding

**Fig. 5:**
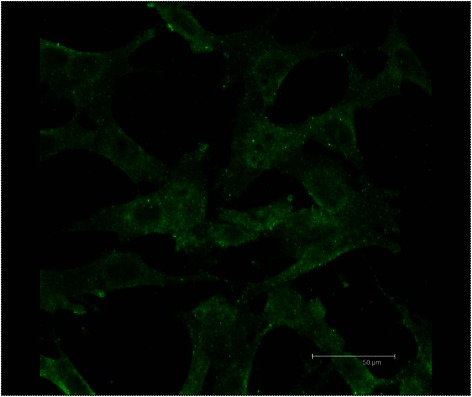
Expression of 14-3-3 sigma in RGC-5 revealed by indirect immunfluorescence. RGC-5 cells were fixed, permeabilised, blocked and incubated with rabbit polyclonal anti 14-3-3 antibodies. Subsequently the cells were incubated with Goat polyclonal secondary antibody to rabbit IgG-H&L conjugated with FITC. The cells were visualized with a fluorescence microscope. The pictures show that 14-3-3 was expressed in all cells and it seems to be distributed in the cytoplasm

**Fig. 6:**
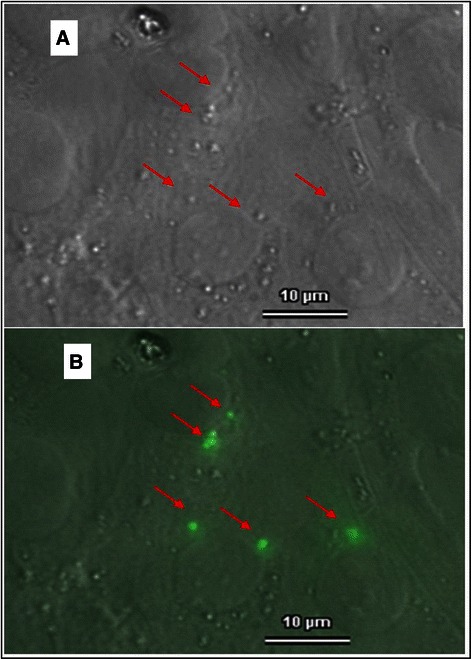
14-3-3 sigma antibody uptake of RGC-5 revealed by indirect immunfluorescence. Living cells were preincubated with polyclonal rabbit anti 14-3-3 antibodies and then fixed, permeabilised, blocked and stained with Goat polyclonal secondary antibody to rabbit IgG-H&L conjugated with FITC. **a**: Bright light microscopy picture. **b**: Corresponding fluorescence micrograph merged with bright light. Red Arrows indicate vesicles, which contain 14-3-3 sigma antibodies, showing antibody uptake into the intact cell

To determine, whether RGC-5 cells express 14-3-3 and were the antibody binds in living cells we performed an indirect immunofluorescence staining. In permeabilised RGC-5 cells we could show binding of the 14-3-3 antibody in the cytoplasm (see Fig. 5). We further investigated the uptake of 14-3-3 antibodies in living RGC-5 cells. We were able to show the uptake of 14-3-3 antibodies in vesicles after preincubation of living RGC-5 for 3 h (Fig. 6).

### Proteomic analysis

**Fig. 7:**
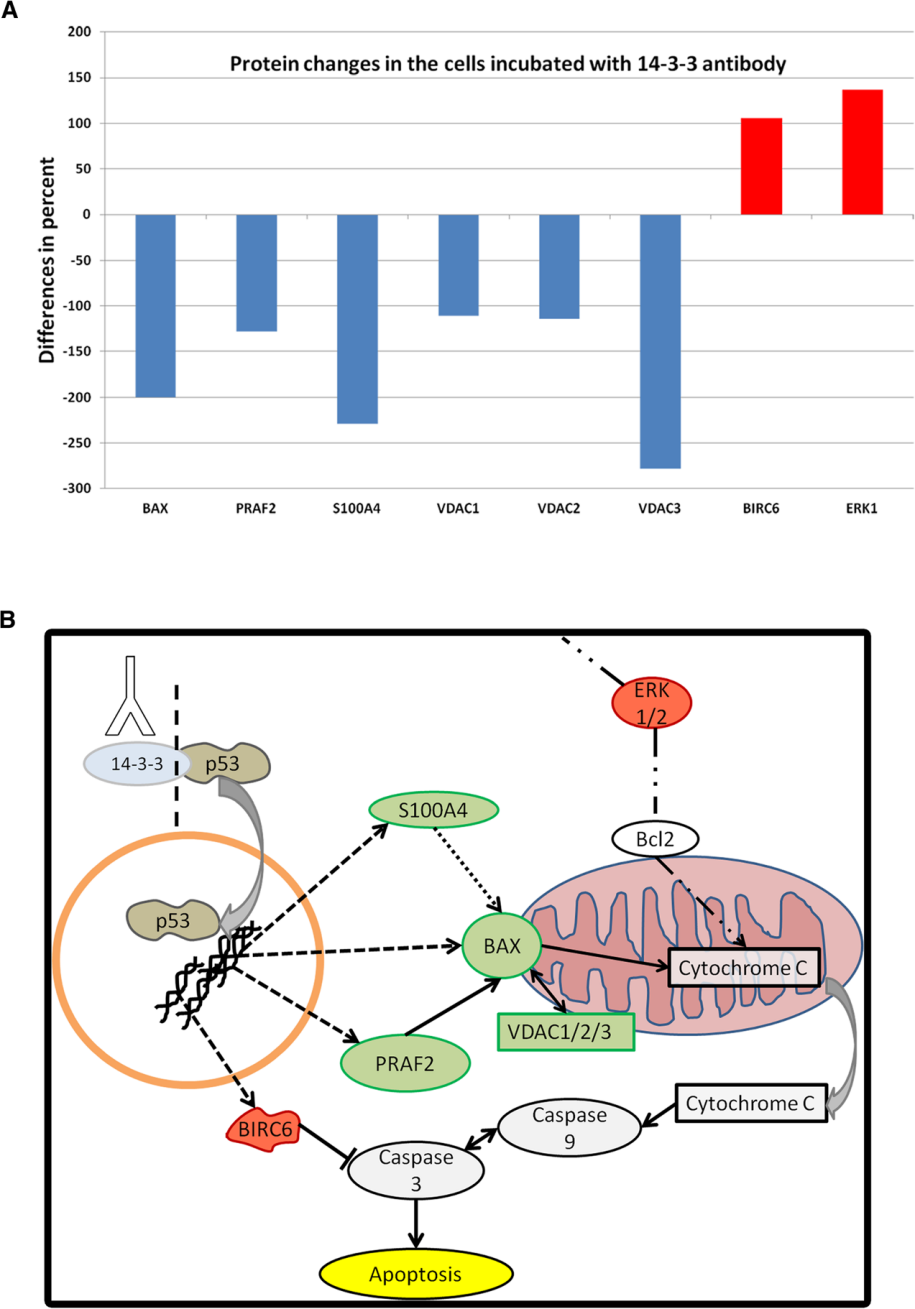
Changed mitochondrial apoptosis pathway in RGC-5 conditioned of treatment with 14-3-3 sigma antibodies. **a**: This graph shows several of the significantly differently regulated proteins in the cells incubated with 14-3-3 antibodies in comparison to control cells. The proteins listed here in some way all are involved in the mitochondrial apoptosis pathway. The changes of the proteins are shown in percent. **b**: This graph schematically visualizes the significantly changed proteins of the mitochondrial apoptosis pathway. Proteins highlighted in green were found to be significantly down-regulated) and proteins highlighted in red significantly up-regulated. The protein 14-3-3 interacts with p53, which is also shown in the graph. It is conceivable that the modulation of 14-3-3 through 14-3-3 sigma antibodies leads to a changed interaction of p53 and thereby to changed expression of p53 target genes such as PRAF2, S100A4 and BAX, which are significantly changed in the cells incubated with 14-3-3 abs. BAX plays a role in releasing cytochrome c from the mitochondrion. Cytochrome C interacts with caspase3, which triggers apoptosis of the cells. It also is conceivable that the interaction of 14-3-3 and STAT3 is altered, which comes to the up-regulation of BIRC6. Another protein which plays also a role in mitochondrial apoptosis is the anti-apoptotic ERK1, which is up-regulated in 14-3-3 sigma treated RGC-5 as well the down-regulation of VDAC 1/2/3

To further investigate the effect of 14-3-3 abs on the protein expression of RGC-5, proteomic analyses were performed. Using a pooled sample, we could identify 1204 proteins of which 225 were significantly differently regulated in cells incubated with 14-3-3 abs (>2 fold increased or < 2 fold decreased) (see Additional file 4: Table S1). The pathway analysis (performed with IPA) showed that many of the differently regulated proteins belong to apoptosis signalling pathways of the cells. We could indicate several changed proteins, such as BAX, BIRC6, PRFA2, S100A4, VDAC 1/2/3 and ERK1, which are involved in the regulation of the mitochondrial apoptosis pathways. BAX, PRFA1, VDAC 1/2/3 and S100A4 were significant down-regulated and BIRC6 and ERK1 were significant up-regulated in cells treated with 14-3-3 abs in comparison to untreated cells (Fig. 7a).

## Discussion

14-3-3 proteins, a group of proteins with many binding partners, play an important role in a wide range of cellular processes and are well known to be altered in many neurodegenerative diseases [31]. In general 14-3-3, a protein kinase inhibitor, has adjudicated anti- apoptotic features. Studies have been able to show the regulator function of 14-3-3 on the MAPK/ERK pathway, protecting cells from apoptosis [32]. The MAPK/ERK pathway can be activated by several extracellular signals and stressors, such as oxidative stress [33], as well as intracellular signals and pathways [34]. In an inactive state 14-3-3 is bound to Raf-1. Briefly, after binding activated Ras and dephosphorylation of S259, 14-3-3 is released from Raf-1 [35]. This evokes activation of the MAPK/ERK pathway with resulting changes of the gene transcription and protein expression, resulting in different cell reactions such as proliferation, but also apoptosis [36]. Elevated levels of 14-3-3 in the cells, as seen in this study, could be an indicator for the activation of the MAPK/ERK pathway. This could be interpreted either that more protective potential is needed in the stressed cells in order to deactivate Raf-1, or possibly we are detecting 14-3-3 after detachment from Raf-1. Other studies using an experimental glaucoma model also showed elevated levels of 14-3-3 protein in the rgc, as well as other significantly changed proteins also found in this study, such as the zinc-finger protein [30]. 14-3-3 proteins are known to be involved in many neurodegenerative diseases. 14-3-3 eta was detected as up-regulated in and around Alzheimer plaques [37], and down-regulated in Lewy bodies in Parkinson’s disease [38]. Another affected pathway, which is linked to 14-3-3 is involved in the calcium homeostasis of cells and therefore is also important for cell survival [39]. Calmodulin, found down-regulated in cells incubated with glaucoma serum, interacts with calcium/calmodulin dependant protein kinase II, which is an N-Methyl-D-aspartate (NMDA) receptor associated protein. The NMDA receptor is thought to play a role in the pathogenesis of glaucoma and in the apoptosis of retinal ganglion cells. A certain level of calcium/calmodulin dependant protein kinase II seems to be important to protect rgc from external factors such as an elevated glutamate level [40] although there is a controversial discussion about the role of up- or down-regulation of calcium/calmodulin dependant protein kinase II when involved in neurodegeneration [41]. Protein kinase C, which is inhibited by 14-3-3, is involved in NMDA receptor trafficking in combination with the activation of the calcium/calmodulin dependant protein kinase II [42].

Clinical and experimental studies show, that autoantibodies are involved in the changes detected in the cells. Several clinical trials show altered autoantibody profiles in the serum of patients suffering from different glaucoma forms (normal tension glaucoma, primary open angle glaucoma) or ocular hypertension [43, 44]. We found that cells react differently depending on the type of glaucoma serum they were incubated with [26]. We found that the antibodies in the serum of glaucoma patients have a large effect of nearly 60 % on the protein profiles of the cells [26]. Glaucoma is not the only neurodegenerative disease with altered autoantibody levels. Studies were able to show a significant down regulation of autoantibodies in the serum of Alzheimer patients in comparison to healthy controls [25, 45].

The effect of one of the down-regulated antibodies was tested within this study and we found that low concentrations of 14-3-3 abs had protective effects on neuroretinal cells.

Our proteomic measurements show that these effects are mediated via the mitochondrial apoptosis pathway. The proteins BAX, BIRC6. PRFA2, S100A4, VDAC 1/2/3 and ERK1 where regulated in a significantly different way in cells incubated with the abs and are involved in the regulation of the intrinsic apoptosis pathway (Fig. 7b). Pro-apoptotic BAX, a member of the Bcl-2 family, was down-regulated in cells incubated with 14-3-3 abs. It plays an important role in the intrinsic apoptotic pathway by binding mitochondrial VDAC which leads to the release of cytochrome c and finally to the initiation of apoptosis [46]. BAX, as well as its transcription factor p53, are associated with neurodegenerative diseases [47]. Deficiency of BAX in DBA/2 J mice protects rgc from cell death [48]. It also is increased in a mouse model of glaucoma [49]. Tumour suppressor p53 is a key regulator of apoptosis and also plays a role in glaucoma [50]. 14-3-3 interacts with p53 and positively regulates its transcriptional activity. It also stabilizes p53 and is involved in the nuclear export and modulation of tumour suppressing activity [51]. Other proteins also were regulated in an anti-apoptotic manner such as S1004 and PRAF2 which were down-regulated in this study. They both can lead to apoptosis [52, 53]. The anti-apoptotic protein BIRC6 belongs to the inhibitor of apoptosis (IAP) family and was up-regulated in cells incubated with 14-3-3 antibodies. BIRC6 is up-regulated in tumours and inhibits active caspase 3 through binding with its BIR domain [54].

Additionally an increased expression of ERK was found in 14-3-3 ab incubated cells. Activated ERK1 is able to phosphorylate many cytoplasmic as well as nuclear targets, which leads to cell proliferation and differentiation [55] and participates in mitochondrial apoptosis [56, 57]. Studies with experimental rat glaucoma models show that the activation of ERK leads to increased survival of rgc after ocular hypertension surgery [58]. Staurosporine leads to changes in the phosphorylation as well as activation status of ERK [59]. The increased ERK expression in the cells incubated with 14-3-3 antibodies could be the link to the protective effect of the 14-3-3 antibody on staurosporine stressed cells. Furthermore, increased ERK1 expression could result in phosphorylation of BAD [60, 61] and provide survival of stressed RGC-5. This could be the mechanism for the increased viability and reduced ROS levels of the cells stressed with H_2_O_2_. Cellular H_2_O_2_ stress induces elevated ROS expression, which increases the expression of JNK-mitogen activated protein kinase (MAPK). JNK itself leads to caspase activation, which contributes to apoptosis of cells [62]. JNK activation also leads to phosphorylation of 14-3-3 and this consequently promotes dephosphorylation of BAD, which has also been shown for retinal ganglion cells [30].

This mechanism was most likely triggered by antibody uptake in vesicles near the nucleus of the cells, as this was detected in the immunohistochemical staining. Antibodies are large proteins with a molecular weight of 140–150 kDa. Antibody uptake of Hsp 27 ab has been demonstrated in retinal ganglion cells [63] and it also was supposed that the antibody can modulate its antigen, by inhibiting the protective function or even inactivating it. In general the mechanisms by which antibodies are transferred into cells, bind to their antigen or translocate into the nucleus or other organelles are not understood very well, one possible mechanism is mediated by myosin 1. Studies show the uptake of anti-DNA antibodies into living cells, mediated by myosin1. The internalized anti-DNA antibodies interact with DNAse in the cytoplasm and inhibit their enzymatic activity. The antibodies were furthermore transferred into the nucleus and recycling to the cell membrane [64].

Patients with other neurodegenerative diseases also show changes in natural autoantibody levels, such as in Alzheimer’s disease, where decreased levels of Aβ-ab are detected, or in Parkinson’s disease, where decreased levels of the antibody against α-synuclein, a protein involved in the pathogenesis of the disease, was found [65]. Studies propose, that decreased levels of the natural autoantibody Aβ-ab in Alzheimer patients lead to a loss of protective effects of the ab and therefore to the deposition of Aβ in the brain [66]. In recent research on a new Alzheimer’s therapy, promising studies have been performed with intravenous Immunoglobulin’s (Ig) showing a reduction of plasma concentration of Aβ1–42 [67].

These results underline our hypothesis, that changes found in the natural autoimmunity of patients suffering from neurodegenerative diseases such as Alzheimer’s or glaucoma have an impact on the pathogenesis of the disease, showing that also the down-regulated autoantibodies have an impact on the regulatory functions and therefore their reduction is most possibly causing vulnerability to damage. We are aware of the fact that RGC-5 cells are not a pure retinal ganglion cell line. Nevertheless, a recently published article summarised the characteristics of RGC-5 and their usage as a cell model line. The authors stated that the majority of the published articles characterising RGC-5 cells showed Brn3, as well as Thy 1 staining, whereas only two papers were not able to detect these markers. Furthermore nestin expression was detected in RGC-5 cells, leading to the conclusion that these cells are of neuronal origin. The authors conclude that RGC-5 cells can act as retinal cell line of neuronal origin in order to follow up initial hypothesis [27].

## Conclusion

In conclusion we were able to show changes in the protein profiles of the cells incubated with POAG serum, especially of proteins involved in regulatory cell mechanisms. In a further step we were able to demonstrate that 14-3-3 abs, which are down-regulated in glaucoma patients have protective effects on RGC-5 cells and are transported into the cells. We were able to detect several proteins changed in an anti-apoptotic manner in those cells incubated with 14-3-3 abs. Altogether these results underline our hypothesis that the changes in the natural autoimmunity found in the serum of patients with the neurodegenerative disease glaucoma play a role in the disease pathogenesis. These results can be seen in accordance with results derived from studies performed for Alzheimer’s disease. We believe that by losing regulatory effects of down-regulated autoantibodies, retinal ganglion cells could become more vulnerable to other stress factors and apoptosis of the cells could be provoked e.g. by an elevated IOP.

## Additional files

Additional file 1: Figure S1. This graph shows the autoantibody levels of the 14-3-3 antibody in the serum of glaucoma patients in comparison to healthy subjects (n = 45). Microarray meassurements of the sera were performed. Glas slides coated with nitrocellulose were spotted with different antigens, also including the 14-3-3 antigen. Then the slides were incubated with the sera of either healthy people or patients suffering from primary open angle glaucoma. The antibodies in the serum bind to their antigen. The bound antibodies are then detected using a secondary antibody to IgG (bound with Cy3). Using a Affymetrix Array scanner, the spots are scanned. The intensity of the bound antibody is analyzed using the intensity of the labelling of the secondary antibody and a comparison between healty and glaucomatous serum is performed. A discrimance analysis was performed in order to detect significant differences. Shown ist the mean of the Intensity (U) with the standard error. We were able to detect a significantly lower level of 14-3-3 antibody in the serum of POAG patients (*p* < 0.01). 
Additional file 2: Figure S2. RGC-5 were preincubated with different anti-myoglobin antibody concentrations and additionally stressed with 20 mM glutamate for 24 h, or 1.5 μM stauorsporine for 5 h. Cell viability was determined using crystal violet and expressed as percent of the control cells + the stress factor (glutamate or staurosporine) (* = *p* < 0.05; ***p* < 0.01). This graph shows the results of the cells stressed with glutamate. No protective effect of the antibody can be seen.
Additional file 3: Figure S3. Non-stressed RGC-5 cells were incubated with the different 14-3-3 ab concentrations (0.05, 0.1, 0.5, 1, 5 and 10 μg/ml). Viability was measured with crystal violet. We were not able to detect any significant changes in the viability of the cells. The graph shows the different cell groups on the X- axis and the viability in % on the Y- axis.
Additional file 4: Table S1 We were able to identify 1204 proteins in the cells. 225 of the proteins were significantly differently regulated in the cells incubated with 14-3-3 antibody in comparison to control cells (>2 fold increased or <2 fold decreased). The significantly changed proteins are listed in Additional file 4: Table S1.

## Abbreviations

Rgc: Retinal ganglion cell
BAX: bcl-2 associated- x-protein
BIRC6: Baculoviral IAP repeat containing 6
S100A4: S100 calcium binding protein A4
PRAF2: PRA1 family protein 2
VDAC 1/2/3: Voltage dependent anion channel
ERK1: Extracellular regulated protein kinase
ROS: Reactive oxygen species
hsp: Heat shock protein
PBS: Phosphate buffered saline, DCFH-DA, 2’,7’-dichlorodihydrofluorescein-diacetate
DCFH: 2’, 7’ dichlorodihydrofluorescein
DCF: 2’,7’-dichlorfluorescein
CDS: Cell dissociation solution
CAN: Acetonitril
AB: Ammoniumbicarbonate
IPA: Ingenuity pathway analysis
IAP: Inhibitor of apoptosis protein
RSK: 90 kDa ribosomal S6 kinase
