# Supplementary figures and images for: Protective effect of 14-3-3 antibodies on stressed neuroretinal cells via the mitochondrial apoptosis pathway
Source: BMC Ophthalmol. 2015 Jun 27;15:64. doi: 10.1186/s12886-015-0044-9 (PMC4482181; doi:10.1186/s12886-015-0044-9)

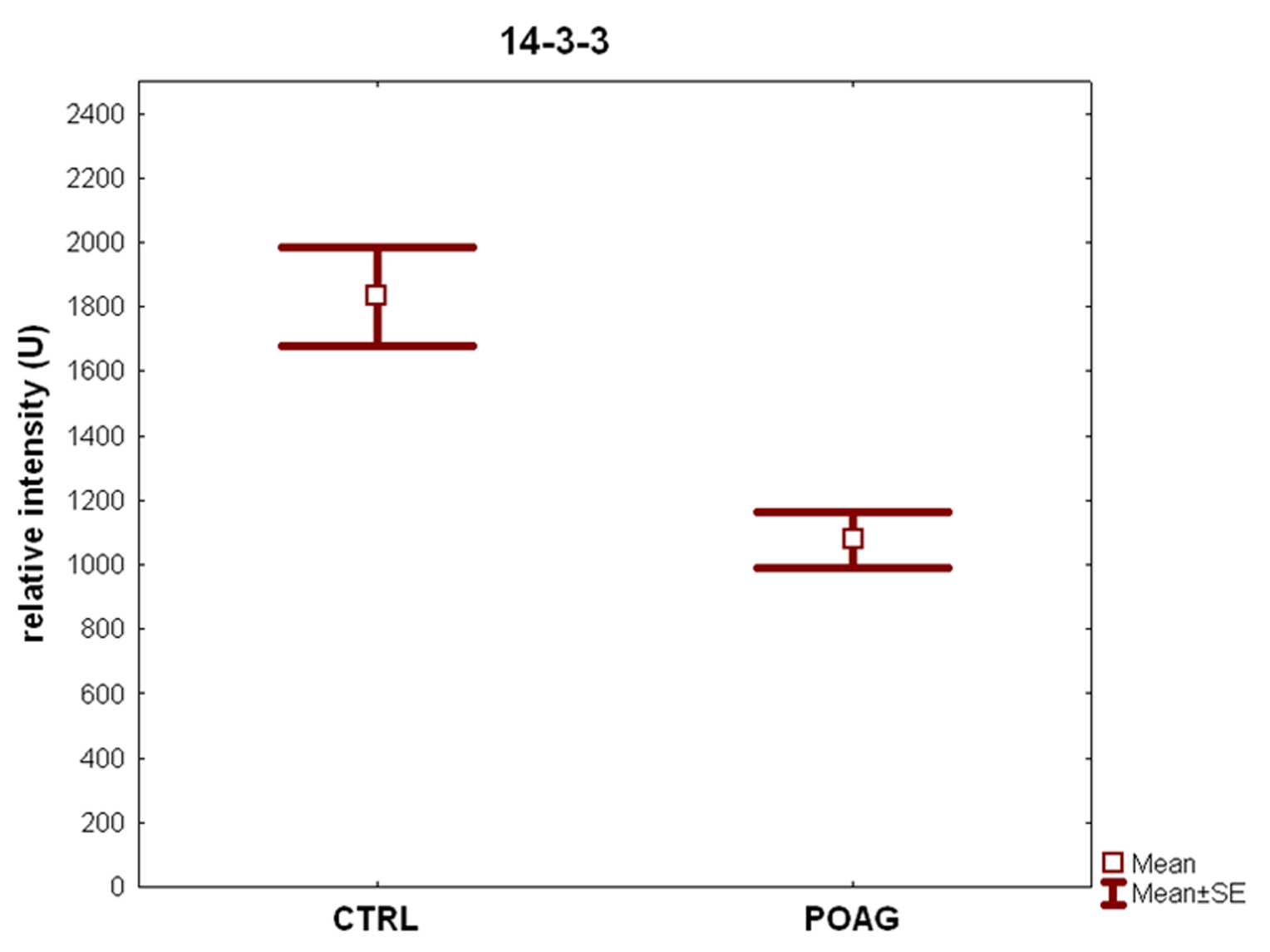

Supplement: Additional file 1: Figure S1. — This graph shows the autoantibody levels of the 14-3-3 antibody in the serum of glaucoma patients in comparison to healthy subjects (n = 45). Microarray meassurements of the sera were performed. Glas slides coated with nitrocellulose were spotted with different antigens, also including the 14-3-3 antigen. Then the slides were incubated with the sera of either healthy people or patients suffering from primary open angle glaucoma. The antibodies in the serum bind to their antigen. The bound antibodies are then detected using a secondary antibody to IgG (bound with Cy3). Using a Affymetrix Array scanner, the spots are scanned. The intensity of the bound antibody is analyzed using the intensity of the labelling of the secondary antibody and a comparison between healty and glaucomatous serum is performed. A discrimance analysis was performed in order to detect significant differences. Shown ist the mean of the Intensity (U) with the standard error. We were able to detect a significantly lower level of 14-3-3 antibody in the serum of POAG patients (p < 0.01). [file 12886_2015_44_MOESM1_ESM.tiff]

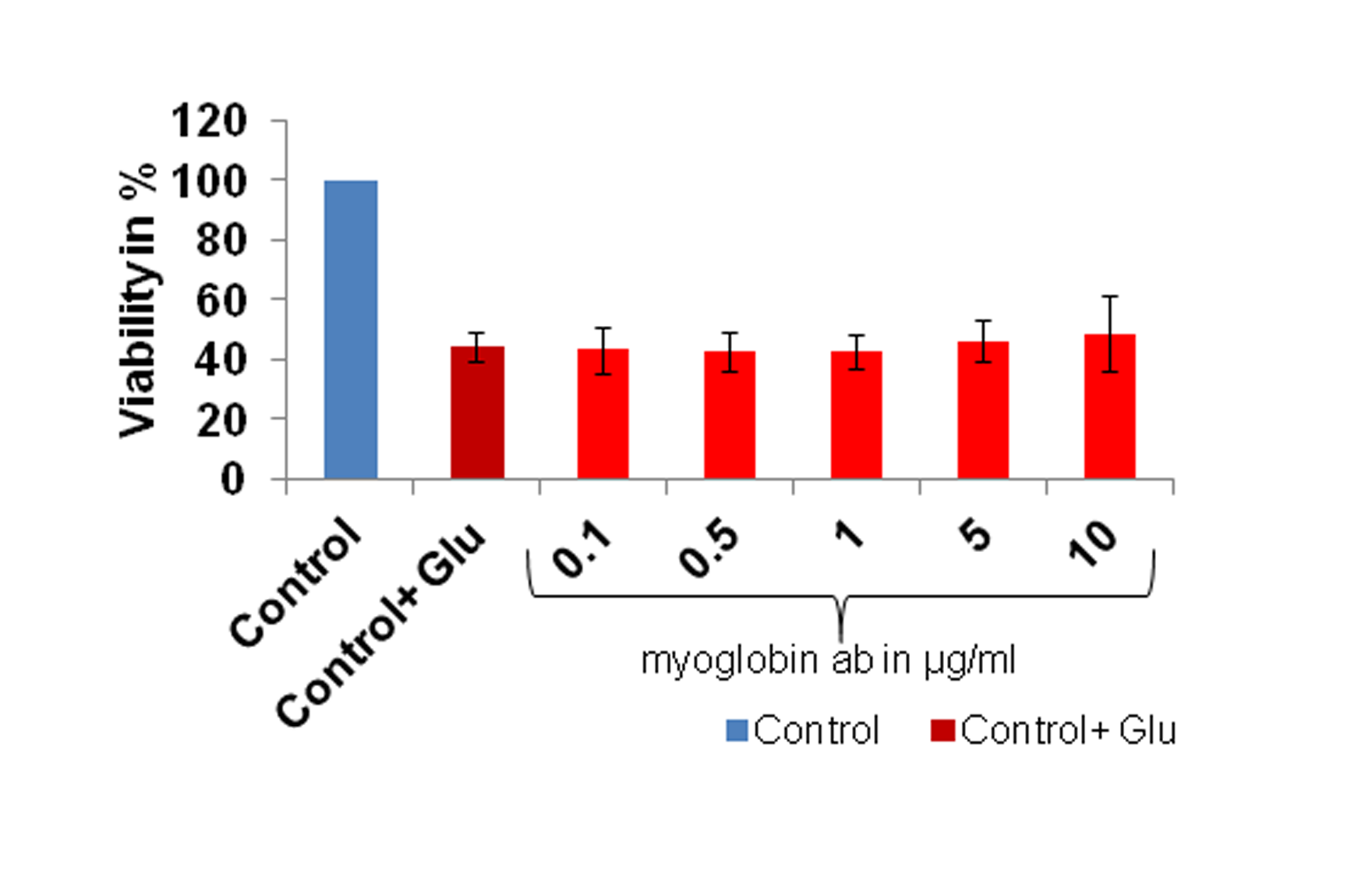

Supplement: Additional file 2: Figure S2. — RGC-5 were preincubated with different anti-myoglobin antibody concentrations and additionally stressed with 20 mM glutamate for 24 h, or 1.5 μM stauorsporine for 5 h. Cell viability was determined using crystal violet and expressed as percent of the control cells + the stress factor (glutamate or staurosporine) (* = p < 0.05; **p < 0.01). This graph shows the results of the cells stressed with glutamate. No protective effect of the antibody can be seen. [file 12886_2015_44_MOESM2_ESM.tiff]

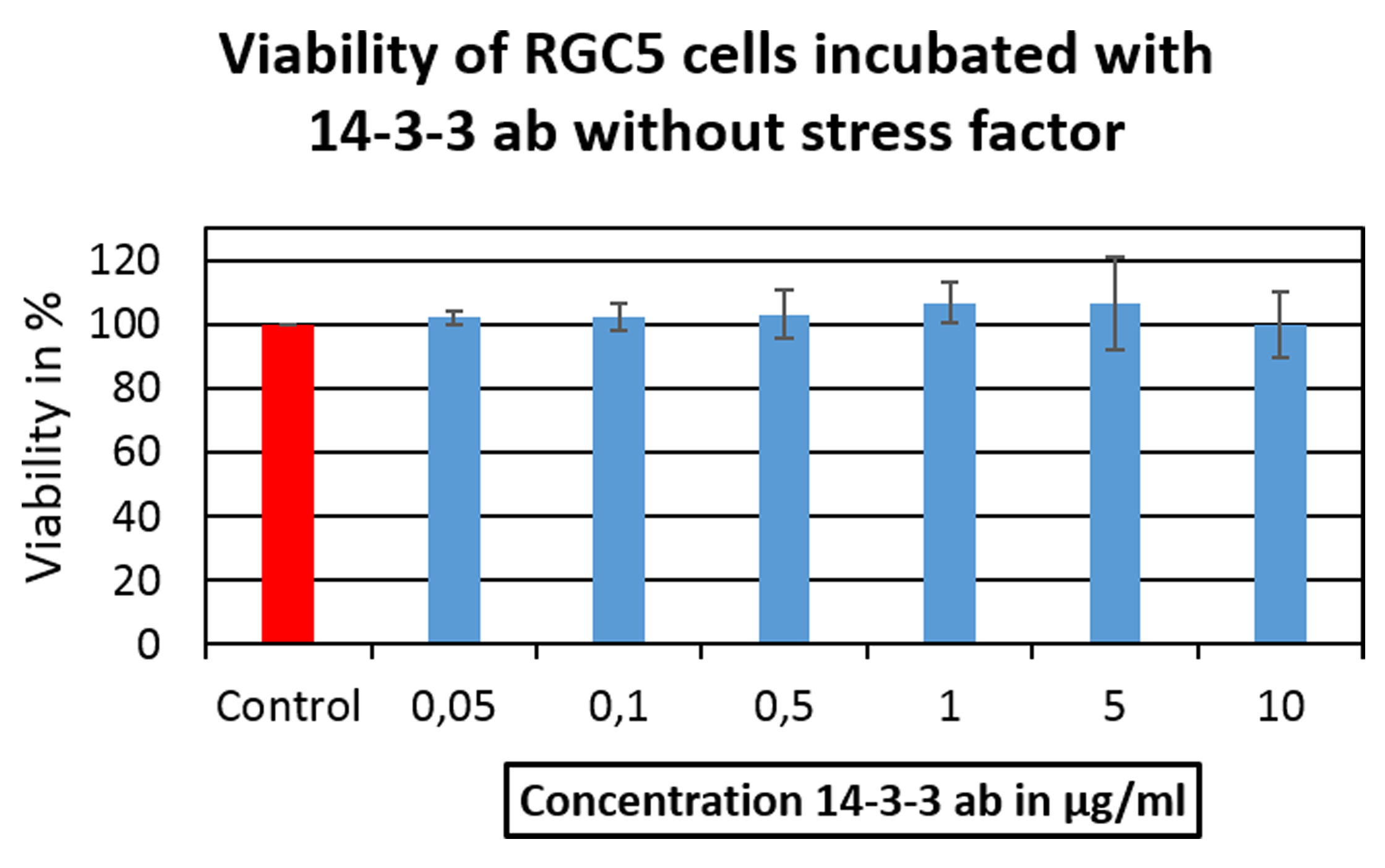

Supplement: Additional file 3: Figure S3. — Non-stressed RGC-5 cells were incubated with the different 14-3-3 ab concentrations (0.05, 0.1, 0.5, 1, 5 and 10 μg/ml). Viability was measured with crystal violet. We were not able to detect any significant changes in the viability of the cells. The graph shows the different cell groups on the X- axis and the viability in % on the Y- axis. [file 12886_2015_44_MOESM3_ESM.tiff]
