# Supplementary material for: Protective effect of 14-3-3 antibodies on stressed neuroretinal cells via the mitochondrial apoptosis pathway
Source: BMC Ophthalmol. 2015 Jun 27;15:64. doi: 10.1186/s12886-015-0044-9 (PMC4482181; doi:10.1186/s12886-015-0044-9)
Supplement: Additional file 4: Table S1 — We were able to identify 1204 proteins in the cells. 225 of the proteins were significantly differently regulated in the cells incubated with 14-3-3 antibody in comparison to control cells (>2 fold increased or <2 fold decreased). The significantly changed proteins are listed in Additional file 4: Table S1. [file 12886_2015_44_MOESM4_ESM.docx]

**Supplement**

Table 1: Significant protein changes in RGC-5 cells upon 14-3-3 sigma antibody treatment

| **ID** | **Entrez Gene Name** | **Location** | **Type(s)** | **Fold Change** |
| --- | --- | --- | --- | --- |
| P02666 | casein beta | Extracellular Space | kinase | -83.858 |
| Q99LS3 | phosphoserine phosphatase | Cytoplasm | phosphatase | -16.839 |
| Q62426 | cystatin B (stefin B) | Cytoplasm | other | -13.045 |
| P20152 | vimentin | Cytoplasm | other | -10.474 |
| Q9QYR9 | acyl-CoA thioesterase 2 | Cytoplasm | enzyme | -9.456 |
| A2AEY2 | four and a half LIM domains 1 | Cytoplasm | other | -8.708 |
| Q9R0P5 | destrin (actin depolymerizing factor) | Cytoplasm | other | -8.045 |
| Q9CR86 | calcium regulated heat stable protein 1. 24kDa | Cytoplasm | other | -7.904 |
| P09671 | superoxide dismutase 2. mitochondrial | Cytoplasm | enzyme | -6.646 |
| P08074 | carbonyl reductase 2 | Cytoplasm | enzyme | -5.71 |
| Q3TTN3 | voltage-dependent anion channel 3 | Cytoplasm | ion channel | -5.561 |
| P62715 | protein phosphatase 2. catalytic subunit. beta isozyme | Cytoplasm | phosphatase | -5.096 |
| Q3SX09 | hemoglobin. beta | Cytoplasm | transporter | -4.944 |
| Q3TW96-1 | UDP-N-acteylglucosamine pyrophosphorylase 1-like 1 | unknown | other | -4.917 |
| Q3TN39 | solute carrier family 3 (activators of dibasic and neutral amino acid transport). member 2 | Plasma Membrane | transporter | -4.916 |
| Q8VDN2 | ATPase. Na+/K+ transporting. alpha 1 polypeptide | Plasma Membrane | transporter | -4.782 |
| P31786 | diazepam binding inhibitor (GABA receptor modulator. acyl-CoA binding protein) | Cytoplasm | other | -4.674 |
| Q05920 | pyruvate carboxylase | Cytoplasm | enzyme | -4.649 |
| P50543 | S100 calcium binding protein A11 | Cytoplasm | other | -4.58 |
| Q62465 | vesicle amine transport protein 1 homolog (T. californica) | Plasma Membrane | transporter | -4.529 |
| O08807 | peroxiredoxin 4 | Cytoplasm | enzyme | -4.395 |
| P08113 | heat shock protein 90kDa beta (Grp94). member 1 | Cytoplasm | other | -4.392 |
| Q564P4 | adenine phosphoribosyltransferase | Cytoplasm | enzyme | -4.356 |
| E9Q9H9 | peroxiredoxin 5 | Cytoplasm | enzyme | -4.31 |
| P13020-1 | gelsolin | Extracellular Space | other | -4.297 |
| P02769 | albumin | Extracellular Space | transporter | -4.296 |
| Q9R1P3 | proteasome (prosome. macropain) subunit. beta type. 2 | Cytoplasm | peptidase | -4.133 |
| P34955 | serpin peptidase inhibitor. clade A (alpha-1 antiproteinase. antitrypsin). member 1 | Extracellular Space | other | -4.122 |
| P35279-1 | RAB6A. member RAS oncogene family | Cytoplasm | enzyme | -3.992 |
| Q07813 | BCL2-associated X protein | Cytoplasm | other | -3.99 |
| E0CXH5 | triosephosphate isomerase 1 | Cytoplasm | enzyme | -3.981 |
| P40142 | transketolase | Cytoplasm | enzyme | -3.939 |
| P23492 | purine nucleoside phosphorylase | Nucleus | enzyme | -3.935 |
| Q9CVB6 | actin related protein 2/3 complex. subunit 2. 34kDa | Cytoplasm | other | -3.879 |
| B1ATE2 | ring finger protein 213 | Plasma Membrane | other | -3.876 |
| P41731 | CD63 molecule | Plasma Membrane | other | -3.847 |
| P20029 | heat shock 70kDa protein 5 (glucose-regulated protein. 78kDa) | Cytoplasm | enzyme | -3.828 |
| Q8VCW8 | acyl-CoA synthetase family member 2 | Cytoplasm | enzyme | -3.691 |
| Q9CQI6 | coactosin-like 1 (Dictyostelium) | Cytoplasm | other | -3.68 |
| Q9D892 | inosine triphosphatase (nucleoside triphosphate pyrophosphatase) | Cytoplasm | enzyme | -3.644 |
| Q922Q8 | leucine rich repeat containing 59 | Cytoplasm | other | -3.511 |
| O88844 | isocitrate dehydrogenase 1 (NADP+). soluble | Cytoplasm | enzyme | -3.497 |
| Q3V2H3 | sorting nexin 12 | unknown | transporter | -3.495 |
| P68037 | ubiquitin-conjugating enzyme E2L 3 | Cytoplasm | enzyme | -3.476 |
| P70202 | latexin | Cytoplasm | other | -3.46 |
| P62075 | translocase of inner mitochondrial membrane 13 homolog (yeast) | Cytoplasm | transporter | -3.456 |
| Q8BP47 | asparaginyl-tRNA synthetase | Cytoplasm | enzyme | -3.435 |
| E9QLV8 | aldehyde dehydrogenase 1 family. member L2 | Cytoplasm | enzyme | -3.414 |
| P84078 | ADP-ribosylation factor 1 | Cytoplasm | enzyme | -3.369 |
| E9Q120 | lon peptidase 1. mitochondrial | Cytoplasm | peptidase | -3.361 |
| P16110 | lectin. galactoside-binding. soluble. 3 | Extracellular Space | other | -3.334 |
| Q9JJU8 | SH3 domain binding glutamic acid-rich protein like | Cytoplasm | other | -3.32 |
| Q05816 | fatty acid binding protein 5 (psoriasis-associated) | Cytoplasm | transporter | -3.258 |
| Q9D1D4-1 | transmembrane emp24-like trafficking protein 10 (yeast) | Cytoplasm | transporter | -3.162 |
| Q9QXS1-13 | plectin | Cytoplasm | other | -3.145 |
| P48678-1 | lamin A/C | Nucleus | other | -3.134 |
| Q9CZD3 | glycyl-tRNA synthetase | Cytoplasm | enzyme | -3.118 |
| Q3UMP2 | 3-hydroxymethyl-3-methylglutaryl-CoA lyase | Cytoplasm | enzyme | -3.11 |
| A8C1V1 | mesencephalic astrocyte-derived neurotrophic factor | Extracellular Space | other | -3.109 |
| P59999 | actin related protein 2/3 complex. subunit 4. 20kDa | unknown | other | -3.09 |
| Q9QYB1 | chloride intracellular channel 4 | Plasma Membrane | ion channel | -3.062 |
| Q8BH95 | enoyl CoA hydratase. short chain. 1. mitochondrial | Cytoplasm | enzyme | -2.979 |
| Q920E5 | farnesyl diphosphate synthase | Cytoplasm | enzyme | -2.969 |
| P70296 | phosphatidylethanolamine binding protein 1 | Cytoplasm | other | -2.931 |
| Q9DCN2-1 | cytochrome b5 reductase 3 | Cytoplasm | enzyme | -2.909 |
| P29391 | ferritin. light polypeptide | Cytoplasm | other | -2.872 |
| Q9DBP5 | cytidine monophosphate (UMP-CMP) kinase 1. cytosolic | Nucleus | kinase | -2.785 |
| P11276 | fibronectin 1 | Extracellular Space | enzyme | -2.751 |
| A6ZI44 | aldolase A. fructose-bisphosphate | Cytoplasm | enzyme | -2.749 |
| Q61792 | LIM and SH3 protein 1 | Cytoplasm | transporter | -2.74 |
| Q9DAS9 | guanine nucleotide binding protein (G protein). gamma 12 | Plasma Membrane | enzyme | -2.712 |
| P07091 | S100 calcium binding protein A4 | Cytoplasm | other | -2.712 |
| Q3TFB5 | N-acetylneuraminic acid synthase | Cytoplasm | enzyme | -2.69 |
| P17742 | peptidylprolyl isomerase A (cyclophilin A) | Cytoplasm | enzyme | -2.69 |
| P62880 | guanine nucleotide binding protein (G protein). beta polypeptide 2 | Plasma Membrane | enzyme | -2.686 |
| P34884 | macrophage migration inhibitory factor (glycosylation-inhibiting factor) | Extracellular Space | cytokine | -2.686 |
| Q7TQI3 | OTU domain. ubiquitin aldehyde binding 1 | unknown | enzyme | -2.679 |
| Q9CZU6 | citrate synthase | Cytoplasm | enzyme | -2.615 |
| O70492 | sorting nexin 3 | Cytoplasm | transporter | -2.613 |
| Q3UG45 | solute carrier family 7 (cationic amino acid transporter. y+ system). member 5 | Plasma Membrane | transporter | -2.612 |
| Q8R1I1 | ubiquinol-cytochrome c reductase. complex III subunit X | Cytoplasm | enzyme | -2.59 |
| Q9CPT4 | chromosome 19 open reading frame 10 | Extracellular Space | cytokine | -2.589 |
| Q9R0P3 | esterase D | Cytoplasm | enzyme | -2.589 |
| Q9DBG9 | Tax1 (human T-cell leukemia virus type I) binding protein 3 | Nucleus | transcription regulator | -2.579 |
| Q9JII6 | aldo-keto reductase family 1. member A1 (aldehyde reductase) | Cytoplasm | enzyme | -2.578 |
| Q9CQA3 | succinate dehydrogenase complex. subunit B. iron sulfur (Ip) | Cytoplasm | enzyme | -2.569 |
| Q9JIG8 | PRA1 domain family. member 2 | unknown | other | -2.564 |
| Q9D358-1 | acid phosphatase 1. soluble | Cytoplasm | phosphatase | -2.559 |
| P45878 | FK506 binding protein 2. 13kDa | Cytoplasm | enzyme | -2.556 |
| Q8K1M6-1 | dynamin 1-like | Cytoplasm | enzyme | -2.536 |
| P12382 | phosphofructokinase. liver | Cytoplasm | kinase | -2.533 |
| P14152 | malate dehydrogenase 1. NAD (soluble) | Cytoplasm | enzyme | -2.515 |
| P00375 | dihydrofolate reductase | unknown | enzyme | -2.494 |
| Q3UKW2 | calmodulin 1 | Nucleus | other | -2.473 |
| P97869 | epoxide hydrolase 1. microsomal (xenobiotic) | Cytoplasm | peptidase | -2.46 |
| Q3UCL0 | annexin A4 | Plasma Membrane | other | -2.442 |
| E9QP98 | serpin peptidase inhibitor. clade B (ovalbumin). member 6 | Cytoplasm | other | -2.437 |
| O35215 | D-dopachrome tautomerase | Cytoplasm | enzyme | -2.436 |
| P16858 | glyceraldehyde-3-phosphate dehydrogenase | Plasma Membrane | enzyme | -2.435 |
| Q9Z2U1 | proteasome (prosome. macropain) subunit. alpha type. 5 | Cytoplasm | peptidase | -2.434 |
| P62245 | ribosomal protein S15a | Cytoplasm | other | -2.427 |
| P42208 | septin 2 | Cytoplasm | enzyme | -2.423 |
| Q3TFD0 | serine hydroxymethyltransferase 2 (mitochondrial) | Cytoplasm | enzyme | -2.403 |
| Q8C2Q7 | heterogeneous nuclear ribonucleoprotein H1 (H) | Nucleus | other | -2.388 |
| Q99LX0 | Parkinson disease (autosomal recessive. early onset) 7 | Nucleus | enzyme | -2.386 |
| P11031 | SUB1 homolog (S. cerevisiae) | Nucleus | transcription regulator | -2.385 |
| P68372 | tubulin. beta 2C | Cytoplasm | other | -2.381 |
| O55023 | inositol(myo)-1(or 4)-monophosphatase 1 | Cytoplasm | phosphatase | -2.374 |
| P19096 | fatty acid synthase | Cytoplasm | enzyme | -2.352 |
| P68369 | tubulin. alpha 1a | Cytoplasm | other | -2.345 |
| P24452 | capping protein (actin filament). gelsolin-like | Nucleus | other | -2.336 |
| Q9CPQ1 | cytochrome c oxidase subunit VIc | Cytoplasm | enzyme | -2.328 |
| Q99PT1 | Rho GDP dissociation inhibitor (GDI) alpha | Cytoplasm | other | -2.32 |
| P10711-1 | transcription elongation factor A (SII). 1 | Nucleus | transcription regulator | -2.298 |
| P08249 | malate dehydrogenase 2. NAD (mitochondrial) | Cytoplasm | enzyme | -2.297 |
| Q60930 | voltage-dependent anion channel 2 | Cytoplasm | ion channel | -2.288 |
| Q99K85 | phosphoserine aminotransferase 1 | Cytoplasm | enzyme | -2.287 |
| Q9CQW2 | ADP-ribosylation factor-like 8B | Plasma Membrane | enzyme | -2.283 |
| Q3TWN8 | aldehyde dehydrogenase 18 family. member A1 | Cytoplasm | kinase | -2.282 |
| P17710-1 | hexokinase 1 | Cytoplasm | kinase | -2.28 |
| P05201 | glutamic-oxaloacetic transaminase 1. soluble (aspartate aminotransferase 1) | Cytoplasm | enzyme | -2.266 |
| O08553 | dihydropyrimidinase-like 2 | Cytoplasm | enzyme | -2.263 |
| P08003 | protein disulfide isomerase family A. member 4 | Cytoplasm | enzyme | -2.262 |
| D4AFX7 | DnaJ (Hsp40) homolog. subfamily C. member 13 | unknown | other | -2.248 |
| P00405 | cytochrome c oxidase subunit II | Cytoplasm | enzyme | -2.245 |
| P97372 | proteasome (prosome. macropain) activator subunit 2 (PA28 beta) | Cytoplasm | peptidase | -2.234 |
| Q60932-1 | voltage-dependent anion channel 1 | Cytoplasm | ion channel | -2.229 |
| Q9DCT8 | cysteine-rich protein 2 | Plasma Membrane | other | -2.224 |
| Q8QZY1 | eukaryotic translation initiation factor 3. subunit L | Cytoplasm | other | -2.206 |
| P47754 | capping protein (actin filament) muscle Z-line. alpha 2 | Cytoplasm | other | -2.183 |
| Q07076 | annexin A7 | Plasma Membrane | ion channel | -2.182 |
| Q9CQ92 | fission 1 (mitochondrial outer membrane) homolog (S. cerevisiae) | Cytoplasm | other | -2.174 |
| Q61598-1 | GDP dissociation inhibitor 2 | Cytoplasm | other | -2.16 |
| Q8R013 | methylenetetrahydrofolate dehydrogenase (NADP+ dependent) 1. methenyltetrahydrofolate cyclohydrolase. formyltetrahydrofolate synthetase | Cytoplasm | enzyme | -2.157 |
| Q3UM23 | ribonuclease/angiogenin inhibitor 1 | Cytoplasm | other | -2.157 |
| P08207 | S100 calcium binding protein A10 | Cytoplasm | other | -2.154 |
| Q9D6F7 | signal sequence receptor. delta (translocon-associated protein delta) | Cytoplasm | other | -2.154 |
| P60710 | actin. beta | Cytoplasm | other | -2.149 |
| O08583-1 | THO complex 4 | Nucleus | transcription regulator | -2.144 |
| P14211 | calreticulin | Cytoplasm | transcription regulator | -2.138 |
| Q62261-1 | spectrin. beta. non-erythrocytic 1 | Plasma Membrane | other | -2.136 |
| Q9CR51 | ATPase. H+ transporting. lysosomal 13kDa. V1 subunit G1 | Cytoplasm | transporter | -2.129 |
| P28352 | APEX nuclease (multifunctional DNA repair enzyme) 1 | Nucleus | enzyme | -2.104 |
| P16045 | lectin. galactoside-binding. soluble. 1 | Extracellular Space | other | -2.067 |
| Q6PGH2 | hematological and neurological expressed 1-like | Cytoplasm | other | -2.065 |
| Q61425 | hydroxyacyl-CoA dehydrogenase | Cytoplasm | enzyme | -2.06 |
| O89086 | RNA binding motif (RNP1. RRM) protein 3 | Nucleus | other | -2.059 |
| E9Q7Q3 | tropomyosin 3 | Cytoplasm | other | -2.032 |
| Q9DBJ1 | phosphoglycerate mutase 1 (brain) | Cytoplasm | phosphatase | -2.026 |
| Q922R8 | protein disulfide isomerase family A. member 6 | Cytoplasm | enzyme | -2.019 |
| Q9ERK4 | CSE1 chromosome segregation 1-like (yeast) | Nucleus | transporter | -2.018 |
| P08228 | superoxide dismutase 1. soluble | Cytoplasm | enzyme | -2.014 |
| Q8BH04 | phosphoenolpyruvate carboxykinase 2 (mitochondrial) | Cytoplasm | kinase | -2.012 |
| P68040 | guanine nucleotide binding protein (G protein). beta polypeptide 2-like 1 | Cytoplasm | enzyme | -2.006 |
| P80313 | chaperonin containing TCP1. subunit 7 (eta) | Cytoplasm | other | 2.009 |
| Q3UEB3-1 | poly-U binding splicing factor 60KDa | Nucleus | other | 2.021 |
| B2RWW6 | GCN1 general control of amino-acid synthesis 1-like 1 (yeast) | Cytoplasm | translation regulator | 2.024 |
| E9PWH9 | ribosomal protein L15 | Cytoplasm | other | 2.024 |
| Q9Z1N5 | DEAD (Asp-Glu-Ala-Asp) box polypeptide 39B | Nucleus | enzyme | 2.04 |
| P14069 | S100 calcium binding protein A6 | Cytoplasm | transporter | 2.062 |
| B8JJL8 | programmed cell death 6 interacting protein | Cytoplasm | other | 2.071 |
| E9PYU6 | baculoviral IAP repeat containing 6 | Cytoplasm | enzyme | 2.111 |
| P32067 | Sjogren syndrome antigen B (autoantigen La) | Nucleus | enzyme | 2.131 |
| E9Q6R7 | utrophin | Plasma Membrane | transmembrane receptor | 2.155 |
| Q9CQR2 | ribosomal protein S21 | Cytoplasm | other | 2.164 |
| Q61205 | platelet-activating factor acetylhydrolase 1b. catalytic subunit 3 (29kDa) | Cytoplasm | enzyme | 2.18 |
| Q921F2 | TAR DNA binding protein | Nucleus | transcription regulator | 2.19 |
| Q9WUK2-1 | eukaryotic translation initiation factor 4H | Cytoplasm | translation regulator | 2.198 |
| O70251 | eukaryotic translation elongation factor 1 beta 2 | Cytoplasm | translation regulator | 2.245 |
| A0PJ96 | microtubule-associated protein 1B | Cytoplasm | other | 2.259 |
| A2AGT5-1 | cytoskeleton associated protein 5 | Nucleus | other | 2.28 |
| Q6ZWV7 | ribosomal protein L35 | Cytoplasm | other | 2.287 |
| Q61990-1 | poly(rC) binding protein 2 | Nucleus | other | 2.299 |
| Q8VDM4 | proteasome (prosome. macropain) 26S subunit. non-ATPase. 2 | Cytoplasm | other | 2.311 |
| Q9EPU0-1 | UPF1 regulator of nonsense transcripts homolog (yeast) | Nucleus | enzyme | 2.354 |
| Q8BNI6 | translocase of outer mitochondrial membrane 70 homolog A (S. cerevisiae) | Cytoplasm | transporter | 2.386 |
| P48024 | eukaryotic translation initiation factor 1 | Cytoplasm | translation regulator | 2.392 |
| Q8VIJ6 | splicing factor proline/glutamine-rich | Nucleus | other | 2.461 |
| Q6ZWY8 | thymosin beta 4. X-linked | Cytoplasm | other | 2.461 |
| Q9D883 | U2 small nuclear RNA auxiliary factor 1 | Nucleus | other | 2.502 |
| Q80V08 | ribosomal protein L17 | Cytoplasm | other | 2.531 |
| E9PXE7 | ribosomal protein S11 | Cytoplasm | other | 2.536 |
| Q9Z2X1-1 | heterogeneous nuclear ribonucleoprotein F | Nucleus | other | 2.608 |
| P46935 | neural precursor cell expressed. developmentally down-regulated 4 | Cytoplasm | enzyme | 2.635 |
| O55135 | eukaryotic translation initiation factor 6 | Cytoplasm | translation regulator | 2.64 |
| P63037 | DnaJ (Hsp40) homolog. subfamily A. member 1 | Nucleus | other | 2.71 |
| Q8VHY0-1 | chondroitin sulfate proteoglycan 4 | Plasma Membrane | other | 2.717 |
| P47963 | ribosomal protein L13 pseudogene 12 | Nucleus | other | 2.722 |
| D3Z3G6 | mitogen-activated protein kinase 3 | Cytoplasm | kinase | 2.723 |
| Q6P8I4 | PEST proteolytic signal containing nuclear protein | Nucleus | other | 2.737 |
| E9QK49 | G1 to S phase transition 1 | Cytoplasm | translation regulator | 2.822 |
| O54734 | dolichyl-diphosphooligosaccharide--protein glycosyltransferase | Cytoplasm | enzyme | 2.877 |
| P62305 | small nuclear ribonucleoprotein polypeptide E pseudogene | Nucleus | other | 2.961 |
| Q61937 | nucleophosmin (nucleolar phosphoprotein B23. numatrin) | Nucleus | transcription regulator | 2.995 |
| Q9EPL8 | importin 7 | Nucleus | transporter | 3.016 |
| P09055 | integrin. beta 1 (fibronectin receptor. beta polypeptide. antigen CD29 includes MDF2. MSK12) | Plasma Membrane | transmembrane receptor | 3.036 |
| P97855 | GTPase activating protein (SH3 domain) binding protein 1 | Nucleus | enzyme | 3.066 |
| Q9WTQ5-1 | A kinase (PRKA) anchor protein 12 | Cytoplasm | transporter | 3.109 |
| Q9JKB3-1 | cold shock domain protein A | Nucleus | transcription regulator | 3.278 |
| Q99M08 | predicted gene 2036 | unknown | other | 3.297 |
| P43276 | histone cluster 1. H1b | Nucleus | other | 3.344 |
| B9EHS6 | mannosidase. alpha. class 2A. member 1 | Cytoplasm | enzyme | 3.348 |
| Q61699-1 | heat shock 105kDa/110kDa protein 1 | Cytoplasm | other | 3.435 |
| Q9D0B6-1 | chromosome X open reading frame 26 | unknown | other | 3.446 |
| P97379-1 | GTPase activating protein (SH3 domain) binding protein 2 | Nucleus | enzyme | 3.53 |
| P63276 | ribosomal protein S17 | Cytoplasm | other | 3.532 |
| P35527 | keratin 9 | Cytoplasm | other | 3.62 |
| P15864 | histone cluster 1. H1c | Nucleus | other | 4.048 |
| E9PW66 | nucleosome assembly protein 1-like 1 | Nucleus | other | 4.155 |
| P43274 | histone cluster 1. H1e | Nucleus | other | 4.185 |
| O54724 | polymerase I and transcript release factor | Nucleus | transcription regulator | 4.269 |
| P46471 | proteasome (prosome. macropain) 26S subunit. ATPase. 2 | Nucleus | peptidase | 4.294 |
| P43275 | histone cluster 1. H1a | Nucleus | other | 4.428 |
| Q9CY58-1 | SERPINE1 mRNA binding protein 1 | Nucleus | other | 4.43 |
| P61290 | proteasome (prosome. macropain) activator subunit 3 (PA28 gamma; Ki) | Cytoplasm | peptidase | 4.75 |
| Q62351 | transferrin receptor (p90. CD71) | Plasma Membrane | transporter | 4.789 |
| Q60865 | cell cycle associated protein 1 | Plasma Membrane | other | 4.997 |
| Q8BH64 | EH-domain containing 2 | Nucleus | other | 5.281 |
| Q99LP6 | GrpE-like 1. mitochondrial (E. coli) | Cytoplasm | other | 5.42 |
| Q9DAW9 | calponin 3. acidic | Cytoplasm | other | 6.006 |
| Q7TPV4 | MYB binding protein (P160) 1a | Nucleus | transcription regulator | 6.067 |
| P52293 | karyopherin alpha 2 (RAG cohort 1. importin alpha 1) | Nucleus | transporter | 6.169 |
| P49817-1 | caveolin 1. caveolae protein. 22kDa | Plasma Membrane | other | 8.403 |
| Q9JIK5 | DEAD (Asp-Glu-Ala-Asp) box polypeptide 21 | Nucleus | enzyme | 17.764 |
| P08071 | lactotransferrin | Extracellular Space | peptidase | 19.498 |
